# Elevated serum uric acid is associated with infertility in women living in America

**DOI:** 10.1038/s41598-023-34702-x

**Published:** 2023-05-11

**Authors:** Xiang Hong, Fanqi Zhao, Wei Wang, Jingying Wu, Xiaoqi Zhu, Bei Wang

**Affiliations:** Key Laboratory of Environmental Medicine and Engineering of Ministry of Education, Department of Epidemiology and Health Statistics, School of Public Health, Southeast University, #87, Dingjiaqiao Road, Gulou District, Nanjing City, 210009 Jiangsu Province China

**Keywords:** Health care, Risk factors

## Abstract

Excessive uric acid levels may affect several organs and systems in the body. There is limited evidence of the effects of high serum uric acid levels on the female reproductive system. This study used the National Health and Nutrition Examination Survey (NHANES) database to explore the relationship between serum uric acid and female infertility. This cross-sectional study included a total of 2197 eligible subjects using data from NHANES 2013-March 2020 pre-pandemic data. Self-reported infertility (ever experiencing an inability to conceive after 12 months of trying to become pregnant) was the main outcome. Logistic regression models and restricted cubic spline were used to analyze the relationship between serum uric acid and female infertility, and stratified analysis was carried out. A total of 295 women self-reported infertility (13.43%). The median uric acid level for all study subjects was 4.4 mg/dL (interquartile range [IQR]: 3.7, 5.1). Serum uric acid levels were higher in the infertility group than in the control group (4.7 mg/dL [IQR: 4.0, 5.3] vs. 4.4 mg/dL [IQR: 3.7, 5.1], *P* < 0.001). After adjusting for age, race, marital status, smoking, alcohol, history of pregnancy, history of diabetes, history of hypertension, fasting glucose, total cholesterol, creatinine in refrigerated serum, low-density lipoprotein cholesterol, direct high-density lipoprotein cholesterol, glycohemoglobin, and body mass index confounders, women with serum uric acid levels at Q3 (4.4–5.1 mg/dL) had a 73% (odds ratio [OR] = 1.73, 95% confidence interval [CI] 1.18, 2.54, *P* = 0.005) higher risk of infertility, and women with uric acid levels at Q4 (5.1–18.0 mg/dL) had an 83% (OR = 1.83, 95% CI 1.22, 2.75, *P* = 0.003) increased risk of infertility compared to women at Q1 (1.6–3.7 mg/dL). The restricted cubic spline also showed that when serum uric acid levels exceeded the reference value, the risk of infertility gradually increased. We also performed a sensitivity analysis based on the complete dataset and found that the results were robust. Higher serum uric acid levels were significantly associated with an increased risk of female infertility. Women planning a pregnancy should have increased serum uric acid monitoring.

## Introduction

Infertility is defined as a failure to conceive after a year or more of regular, unprotected sex^1^. Studies have shown that infertility may affect up to 15% of couples of childbearing age^2^. The prevalence of infertility has been growing at an alarming rate, affecting more and more people worldwide^3^. Infertility not only causes physical and mental harm to couples but also has a negative impact on fertility rates in many countries. It has been reported that 33–41% of infertility is caused by female factors only^1^, and the most common causes include ovulation dysfunction and fallopian tube disease^4^. In addition, lifestyle and environmental factors as well as endocrine diseases, such as polycystic ovary syndrome and endometriosis, may also lead to female infertility.

Uric acid is the final product of purine metabolism^5^. Studies have shown that uric acid at normal physiological levels has protective antioxidant effects^6^, accounting for two thirds of the total antioxidant capacity of plasma^7^, and it is an important antioxidant organic compound^8^. With the development of the economy and improving living standards, the dietary structures of modern people have undergone tremendous changes. The availability of beef, lamb, and seafood has increased purine intake and uric acid levels, leading to a significant increase in the incidence of hyperuricemia and gout in the population^9^. In addition, several studies have found that high uric acid levels are also strongly associated with metabolic syndrome and cardiovascular disease^10–13^.

Studies have explored the influence of uric acid on male semen quality and found that high serum uric acid levels may lead to sperm motility defects and a decline in vitality^14^ via changes in reproductive hormone levels and oxidative stress^15^, thereby affecting male reproductive health^16^. However, there are relatively few studies on the relationship between uric acid and the female reproductive system. Although some studies have explored the possible mechanisms of uric acid in the development of polycystic ovary syndrome^17^ and endometriosis^18,19^, direct evidence for a relationship between uric acid and female infertility is still unclear. Therefore, we used data in the National Health and Nutrition Examination Survey (NHANES) database to analyze the relationship between serum uric acid and female infertility, providing a reference for the prevention and treatment of infertility.

## Materials and methods

### Data source and study population

**Figure 1:**
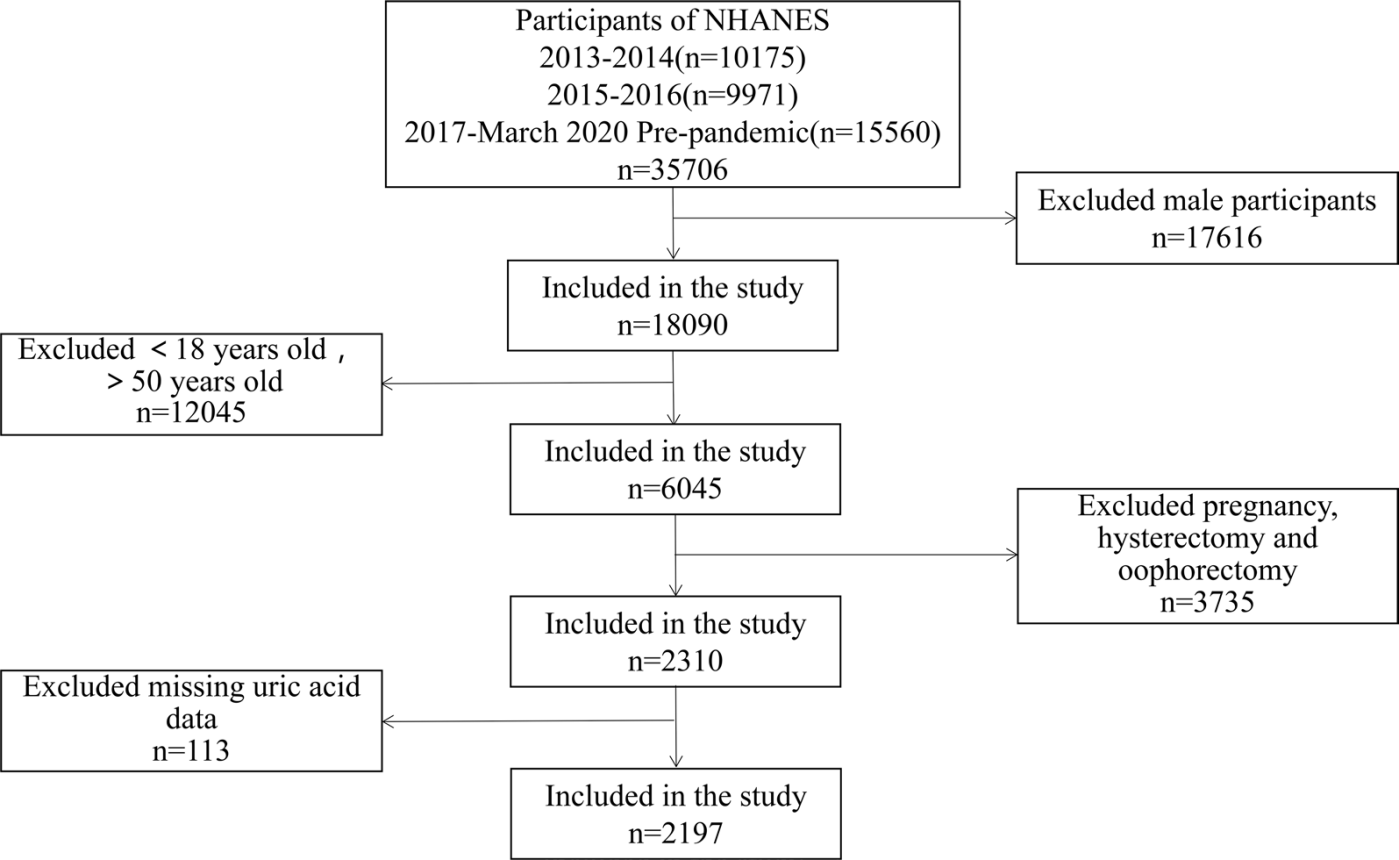
Screening flow chart of research participants.

The NHANES is a major program of the National Center for Health Statistics (NCHS) that is designed to assess the health and nutritional status of adults and children in the United States. It obtains individual health data through interviews, physical measurements, laboratory examinations, etc. The NHANES database is updated every 2 years. Due to the COVID-19 pandemic, on-site operations were suspended in March 2020, and the 2020 survey was not completed. Therefore, data from 2017 to 2018 were combined with data from 2019 to March 2020. We selected the sample (n = 35,706) from NHANES 2013-March 2020 pre-pandemic data. From the 35,706 subjects, we excluded males (n = 17,616), those younger than 18 years or older than 50 years (n = 12,045), pregnant women or those who had undergone a hysterectomy or oophorectomy (n = 3735), and those for whom no uric acid data was available (n = 113). Therefore, 2197 eligible subjects were included in our analysis (Fig. 1).

### Study variables

For laboratory data, the NHANES includes a method file for each variable. According to the NHANES laboratory method document, serum uric acid concentrations were measured using a colorimetric method, and the normal range of serum uric acid values in adult women was 2.4–5.7 mg/dL. Data on the covariates fasting blood glucose, total cholesterol, serum creatinine, low-density lipoprotein (LDL) cholesterol, high-density lipoprotein (HDL) cholesterol and glycosylated hemoglobin were also measured according to their corresponding methods^20^.

The outcome variable ‘infertility’ was obtained by asking whether the subject had tried to become pregnant for at least 1 year but was not pregnant. Women who answered ‘yes’ were classified as infertile, and this definition has been widely used in other studies^21–23^. Other covariates, such as age, race, marriage and childbearing history, smoking history, drinking history, pregnancy history, diabetes history, and hypertension history, were also obtained through the NHANES questionnaire.

More information about variables can be found at https://www.cdc.gov/nchs/nhanes/index.htm.

### Statistical analysis

The continuous variable ‘serum uric acid value’ was classified into three categories according to its normal range (2.4–5.7 mg/dL) and four categories according to its quartile (Q1: 1.6–3.7, Q2: 3.7–4.4, Q3: 4.4–5.1, Q4: 5.1–18.0). Categorical variables are reported as n (percentage), and continuous variables are reported as mean (standard deviation) and median (interquartile interval). Multiple imputation was performed on missing values. Logistic regression models were used to analyze the relationship between serum uric acid and female infertility. Three models were established: an unadjusted model, a partially adjusted model (adjusted for age, race, marital status, smoking, alcohol, history of pregnancy, history of diabetes, history of hypertension), and a fully adjusted model (additionally adjusted for fasting glucose, total cholesterol, creatinine in refrigerated serum, LDL-cholesterol, direct HDL-cholesterol, glycohemoglobin, and body mass index [BMI]). Stratified analysis was conducted for age, marital status, and BMI. As NHANES measured uric acid differently before and after 2017 and specifies different ranges of normal values, we also stratified the analysis for the years in which the data were collected. A restricted cubic spline was drawn to solve the nonlinear relationship between serum uric acid and infertility. All statistical analyses were performed using R 4.1.1, and results with *P* values less than 0.05 were considered statistically significant.

### Ethics approval and consent to participate

Centers for Disease Control and Prevention research on human participants complies with the Health and Human Services Policy for Protection of Human Research Subjects. All National Health and Nutrition Examination Survey procedures and protocols have been reviewed and approved by the National Center for Health Statistics Research Ethics Review Board.

## Results

### Baseline characteristics

The demographic characteristics and health-related information of the study subjects are shown in Table 1. Among 2197 eligible subjects, 295 women suffered from infertility. The average age of all subjects was 34.33 years (standard deviation: 6.67), and the difference between the average ages of the infertility group and the control group was statistically significant (35.39 ± 6.14 vs. 34.17 ± 6.73, *P* = 0.003). The ethnicities of the research subjects included Mexican American, other Hispanic, non-Hispanic white, non-Hispanic Black, non-Hispanic Asian, and other race, of which non-Hispanic white accounted for the largest proportion. There were also statistical differences in marital status, glycohemoglobin, and BMI between the infertility group and the control group (*P* < 0.05). When uric acid was used as a continuous variable, the median serum uric acid level was 4.7 (IQR: 4.0, 5.3) for women in the infertility group and 4.4 (IQR: 3.7, 5.1) for those in the control group, with a statistically significant difference between the two groups. Serum uric acid levels were also statistically different between women in the infertility and control groups when uric acid was categorized into four categorical variables according to quartile spacing.

**Table 1:** Basic demographic and biological characteristics of infertile females and controls participating in NHANES 2013–2020.

	Total	Infertility	Control	*P* value
Subjects (%)	2197 (100)	295 (13.43)	1902 (86.57)	
Age (years) [mean (SD)]	34.33 (6.67)	35.39 (6.14)	34.17 (6.73)	**0.003**
Race (%)				0.139
Mexican American	408 (18.6)	43 (14.6)	365 (19.2)	
Other Hispanic	252 (11.5)	30 (10.2)	222 (11.7)	
Non-Hispanic White	653 (29.7)	106 (35.9)	547 (28.8)	
Non-Hispanic Black	546 (24.9)	69 (23.4)	477 (25.1)	
Non-Hispanic Asian	223 (10.2)	30 (10.2)	193 (10.1)	
Other Race—Including Multi-Racial	115 (5.2)	17 (5.8)	98 (5.2)	
Marital status (%)				**0.001**
Married/Living with Partner	1431 (65.1)	216 (73.2)	1215 (63.9)	
Widowed/Divorced/Separated	278 (12.7)	38 (12.9)	240 (12.6)	
Never married	488 (22.2)	41 (13.9)	447 (23.5)	
Smoking (%)				0.730
Yes	493 (22.4)	69 (23.4)	424 (22.3)	
No	1704 (77.6)	226 (76.6)	1478 (77.7)	
Alcohol (%)				1.000
Yes	19 (0.9)	3 (1.0)	16 (0.8)	
No	2178 (99.1)	292 (99.0)	1886 (99.2)	
History of pregnancy (%)				
Yes	2197 (100.0)	295 (100.0)	1902 (100.0)	
No	0 (0.00)	0 (0.00)	0 (0.00)	
History of diabetes (%)				0.095
Yes	108 (4.9)	22 (7.5)	86 (4.5)	
No	2052 (93.4)	268 (90.8)	1784 (93.8)	
Borderline	37 (1.7)	5 (1.7)	32 (1.7)	
History of hypertension (%)				0.202
Yes	357 (16.2)	58 (19.7)	299 (15.7)	
No	1838 (83.7)	237 (80.3)	1601 (84.2)	
Unknown	2 (0.1)	0 (0.00)	2 (0.1)	
Fasting glucose (mmol/L)				0.628
Median (IQR)	5.33 (5.05, 5.71)	5.30 (4.94, 5.77)	5.33 (5.05, 5.66)	
Missing (%)	1147 (52.21)	161 (54.58)	986 (51.84)	
Total Cholesterol (mg/dL)				0.106
Median (IQR)	174.00 (155.00, 199.00)	178.00 (157.25, 203.00)	174.00 (154.00, 198.00)	
Missing (%)	1 (0.05)	1 (0.34)	0 (0.00)	
Creatinine, refrigerated serum (mg/dL)				0.075
Median (IQR)	0.69 (0.62, 0.79)	0.71 (0.63, 0.80)	0.69 (0.62, 0.79)	
Missing (%)	1 (0.05)	0 (0.00)	1 (0.05)	
LDL-cholesterol (mg/dL)				0.184
Median (IQR)	101.00 (82.00, 120.00)	101.00 (85.50, 121.50)	101.00 (81.00, 120.00)	
Missing (%)	1167 (53.12)	164 (55.59)	1003 (52.73)	
Direct HDL-cholesterol (mg/dL)				0.303
Median (IQR)	53.00 (44.00, 64.00)	52.00 (42.25, 63.00)	53.00 (44.00, 64.00)	
Missing (%)	1 (0.05)	1 (0.34)	0 (0.00)	
Glycohemoglobin (%)				**0.010**
Median (IQR)	5.30 (5.10, 5.60)	5.40 (5.10, 5.70)	5.30 (5.10, 5.60)	
Missing (%)	2 (0.09)	0 (0.00)	2 (0.11)	
BMI (continuous variable)				**0.004**
Median (IQR)	28.80 (23.90, 34.95)	30.45 (24.60, 37.03)	28.60 (23.90, 34.50)	
Missing (%)	14 (0.64)	1 (0.34)	13 (0.68)	
BMI (categorical variable) (%)				**0.014**
< 18.5	42 (1.9)	4 (1.4)	38 (2.0)	
18.5–24.9	614 (28.1)	77 (26.2)	537 (28.4)	
25–29.9	548 (25.1)	57 (19.4)	491 (26.0)	
≥ 30	979 (44.8)	156 (53.1)	823 (43.6)	
Uric acid (continuous variable) (mg/dL)				** < 0.001**
Median (IQR)	4.40 (3.70, 5.10)	4.70 (4.00, 5.30)	4.40 (3.70, 5.10)	
Missing (%)	0 (0.00)	0 (0.00)	0 (0.00)	
Uric acid (categorical variable) (%)				0.233
2.4–5.7	1903(86.6)	249(84.4)	1654(87.0)	
< 2.4	17(0.8)	1(0.3)	16(0.8)	
> 5.7	277(12.6)	45(15.3)	232(12.2)	
Uric acid (quartile) (%)				** < 0.001**
Q1 (1.6–3.7)	560 (25.5)	52 (17.6)	508 (26.7)	
Q2 (3.7–4.4)	570 (25.9)	67 (22.7)	503 (26.4)	
Q3 (4.4–5.1)	527 (24.0)	84 (28.5)	443 (23.3)	
Q4 (5.1–18.0)	540 (24.6)	92 (31.2)	448 (23.6)	

*LDL-cholesterol* low-density lipoprotein cholesterol, *direct HDL-cholesterol* direct high-density lipoprotein cholesterol, *BMI* body mass index. Significant values are in [bold].

### Associations between uric acid and infertility

When we analyzed uric acid as a continuous variable, we found that the risk of female infertility increased with increasing serum uric acid levels in all three models (unadjusted model: OR = 1.24, 95% CI [1.11, 1.37], *P* < 0.001; partially adjusted model: OR = 1.22, 95% CI [1.1, 1.36], *P* < 0.001; fully adjusted model: OR = 1.21, 95% CI [1.07, 1.36], *P* = 0.002). After converting uric acid from a continuous variable to a quadratic variable (quartiles), Q3 and Q4 showed a significant positive association with infertility compared to Q1 in all three models. In the fully adjusted model, the risk of infertility was 1.73 times greater in women with serum uric acid levels in Q3 than in Q1 (OR = 1.73, 95% CI [1.18, 2.54], *P* = 0.005) and 1.83 times greater in women in Q4 than in Q1 (OR = 1.83, 95% CI [1.22, 2.75], *P* = 0.003) (Table 2).

**Table 2:** Unadjusted and adjusted odds ratios (ORs) for serum uric acid levels and infertility risk.

	Infertility	Control	Unadjusted OR (95%CI)	*P* value	Adjusted OR (95%CI)^a^	*P* value	Adjusted OR (95%CI)^b^	*P* value
Uric acid (continuous variable)	295	1902	**1.24 (1.11, 1.37)**	** < 0.001**	**1.22 (1.1, 1.36)**	** < 0.001**	**1.21 (1.07, 1.36)**	**0.002**
Uric acid (categorical variable)								
2.4–5.7	249	1654	1.00 (1.00, 1.00)	1	1.00 (1.00, 1.00)	1	1.00 (1.00, 1.00)	1
< 2.4	1	16	0.42 (0.05, 3.14)	0.395	0.38 (0.05, 2.9)	0.35	0.45 (0.06, 3.48)	0.446
> 5.7	45	232	1.29 (0.91, 1.82)	0.151	1.2 (0.84, 1.71)	0.319	1.1 (0.75, 1.6)	0.634
Uric acid (quartile)								
Q1 (1.6–3.7)	52	508	1.00 (1.00, 1.00)	1	1.00 (1.00, 1.00)	1	1.00 (1.00, 1.00)	1
Q2 (3.7–4.4)	67	503	1.3 (0.89, 1.91)	0.177	1.29 (0.88, 1.9)	0.198	1.24 (0.84, 1.83)	0.282
Q3 (4.4–5.1)	84	443	**1.85 (1.28, 2.68)**	**0.001**	**1.85 (1.27, 2.68)**	**0.001**	**1.73 (1.18, 2.54)**	**0.005**
Q4 (5.1–18.0)	92	448	**2.01 (1.4, 2.88)**	** < 0.001**	**1.98 (1.37, 2.88)**	** < 0.001**	**1.83 (1.22, 2.75)**	**0.003**

^a^Adjusted for age, race, marital status, smoking, alcohol, history of pregnancy, history of diabetes, history of hypertension.

^b^Adjusted for age, race, marital status, smoking, alcohol, history of pregnancy, history of diabetes, history of hypertension, fasting glucose, total cholesterol, creatinine-refrigerated serum, LDL-cholesterol, direct HDL-cholesterol, glycohemoglobin, BMI. Significant values are in [bold].

### Non-linear relationship between uric acid and infertility

**Figure 2:**
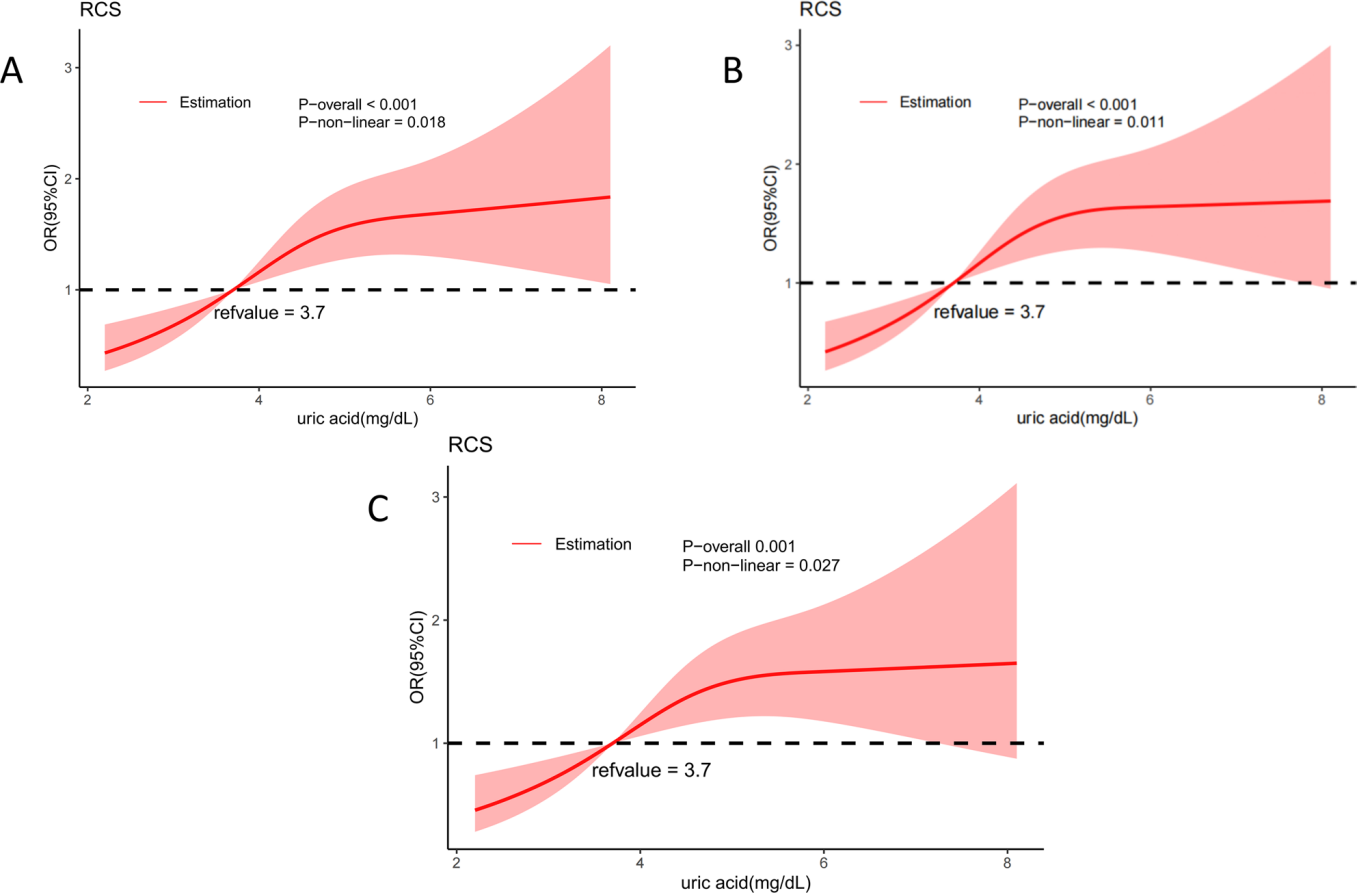
Restricted cubic spline between serum uric acid and infertility risk. (**A**) Unadjusted model. (**B**) Model adjusted for age, race, marital status, smoking, alcohol, history of pregnancy, history of diabetes, history of hypertension. (**C**) Model based on B additionally adjusted for fasting glucose, total cholesterol, creatinine-refrigerated serum, low-density lipoprotein cholesterol, direct high-density lipoprotein cholesterol, glycohemoglobin, and body mass index.

In the three non-linear models, the *P* values for serum uric acid and infertility were less than 0.05, indicating a nonlinear correlation. As shown in Fig. 2, the risk of female infertility increased with increases in serum uric acid levels. Using the 25th percentile of uric acid (3.7 mg/dL) as the reference value, serum uric acid values higher than 3.7 mg/dL increased the risk of female infertility. As uric acid values reached 5.5 mg/dL, the risk of infertility gradually stabilized.

### Stratified analysis

Covariates with statistical differences at baseline were stratified (Table 3). We found that among women ≤ 35 years, those with serum uric acid levels at Q3 (OR = 2.01, 95% CI [1.07, 3.78], *P* = 0.03) and Q4 (OR = 2.47, 95% CI [1.29, 4.73], *P* = 0.006) had a higher risk of infertility compared to those with serum uric acid levels at Q1. Similar results were observed among women who were married or living with their partners (Q3: OR = 2, 95% CI [1.25, 3.19], *P* = 0.004; Q4: OR = 1.84, 95% CI [1.12, 3.04], *P* = 0.016). BMI was stratified according to international standards. In the normal range of BMI (18.5–23.9), it was found that women in Q3 were more likely to suffer from infertility than those in Q1 (OR = 2.27, 95% CI [1.13, 4.56], *P* = 0.021). In overweight (BMI: 25–29.9) and obese (BMI: ≥ 30) women, serum uric acid levels at Q4 were associated with a higher risk of infertility (overweight: OR = 2.51, 95% CI [1.08, 5.83], *P* = 0.033; obese: OR = 1.85, 95% CI [1.01, 3.4], *P* = 0.048). In the 2013–2016 data, the association of infertility with serum uric acid levels in Q3 and Q4 was statistically significant (Q3: OR = 2.25, 95% CI [1.3, 3.87], *P* = 0.004; Q4: OR = 2.08, 95% CI [1.17, 3.69], *P* = 0.013). Although the results for some subgroups were not statistically significant in stratified analyses, the point estimates of OR values did not change, the intervals only became wider, which may be due to the small sample size.

**Table 3:** Stratified analysis of uric acid and infertility risk based on age, marital status, BMI, and database year.

Stratified factor	N	Q1	Q2 OR (95% CI)	*P*	Q3 OR (95% CI)	P	Q4 OR (95% CI)	*P*
Age (years)								
≤ 35	1135	Ref	1.63 (0.86, 3.09)	0.132	**2.01 (1.07, 3.78)**	**0.03**	**2.47 (1.29, 4.73)**	**0.006**
> 35	1062	Ref	1.04 (0.63, 1.73)	0.873	1.59 (0.95, 2.63)	0.075	1.44 (0.84, 2.47)	0.191
Marital status								
Married/Living with Partner	1431	Ref	1.41 (0.88, 2.24)	0.152	**2 (1.25, 3.19)**	**0.004**	**1.84 (1.12, 3.04)**	**0.016**
Widowed/Divorced/Separated	278	Ref	0.9 (0.3, 2.7)	0.852	1.16 (0.39, 3.48)	0.793	2.07 (0.69, 6.24)	0.196
Never married	488	Ref	0.84 (0.28, 2.5)	0.748	1.24 (0.43, 3.59)	0.685	1.32 (0.45, 3.92)	0.614
BMI								
< 18.5	42	Ref	–	–	–	–	–	–
18.5–24.9	616	Ref	1.55 (0.81, 2.96)	0.185	**2.27 (1.13, 4.56)**	**0.021**	1.0043 (0.365, 2.7632)	0.993
25–29.9	553	Ref	0.79 (0.33, 1.89)	0.59	1.43 (0.61, 3.35)	0.409	**2.51 (1.08, 5.83)**	**0.033**
≥ 30	986	Ref	1.3 (0.66, 2.54)	0.448	1.83 (0.98, 3.4)	0.058	**1.85 (1.01, 3.4)**	**0.048**
Database year								
2013–2016	1266	Ref	1.54 (0.89, 2.67)	0.123	**2.25 (1.3, 3.87)**	**0.004**	**2.08 (1.17, 3.69)**	**0.013**
2017-March 2020 pre-pandemic	931	Ref	0.97 (0.54, 1.75)	0.925	1.32 (0.74, 2.35)	0.35	1.74 (0.95, 3.2)	0.072

*BMI* body mass index. Significant values are in [bold].

### Complete case analysis

We re-analyzed the raw data without imputing missing values and found that it was not significantly different from the main results. Both Q3 and Q4 of serum uric acid levels showed a positive association with infertility in women (Supplementary Tables 1 and 2).

## Discussion

In this study, we analyzed the association between female infertility and serum uric acid using data from NHANES 2013 to March 2020 pre-pandemic. The results showed that there was a positive correlation between higher serum uric acid levels and female infertility. Compared with those in Q1, women in Q3 and Q4 had a higher risk of infertility. We showed a non-linear association between serum uric acid levels and infertility by plotting cubic spline plots, and overall, the risk of infertility increased with uric acid levels.

Few previous studies have examined the relationship between serum uric acid and female infertility. It was reported that reproductive hormones such as estradiol^24,25^ and testosterone^26^ can affect serum uric acid levels. However, the effect of uric acid on reproductive hormones has not been clarified^27^. In addition, a higher level of uric acid is associated with an increased probability of anovulatory disorder^28^. Uric acid is a purine derivative, and purine may inhibit the maturation of oocytes^29–32^. High uric acid levels may cause damage to the female reproductive system by inducing oxidative stress^33^, abnormal lipid metabolism^34^, and systemic aseptic inflammation^18^. Some studies have reported that increased serum uric acid levels aggravate metabolic abnormalities related to polycystic ovary syndrome^17,35,36^. The risk of hyperandrogenism in polycystic ovary syndrome patients increases with increasing serum uric acid levels^37^, and excessive androgens lead to a large amount of follicular atresia in women, eventually leading to ovulation disorders^38^. Interleukin-1β produced when high levels of uric acid trigger an inflammatory^18^ reaction may induce ovulation and inhibit endometrial metaplasia, which may affect female embryo implantation, leading to infertility^39^.

The current range of normal values for serum uric acid does not take into account its effect on infertility. Our study found no meaningful results when the normal value range was used to classify uric acid using three categories. However, when categorization was performed using quartiles, statistically significant associations were shown between uric acid levels and increased risk of infertility in Q3 (4.4–5.1) and Q4 (5.1–18.0). These results suggest that the cut-off values for healthy serum uric acid levels should be revisited.

Our research has a number of strengths. We used data from the NHANES, whose benefits include a large sample size, detailed content, wide coverage, standardized implementation, and wide representation. For the non-linear relationship between serum uric acid and infertility, we conducted logistic regression analysis, drew a restricted cubic spline, and conducted stratified analysis for covariates with statistical differences between infertility patients and the control group. In addition, in order to limit the influence of confounding factors, we established three adjustment models. However, our research also has limitations. First, our study was cross-sectional and could not show a causal association between serum uric acid levels and female infertility. Second, our sample size was not adequate, especially for performing stratified analyses. Third, although we adjusted our model, there may still be unmeasured confounding factors, as infertility is a complex problem with many potential confounders. Finally, the NHANES offers a one-time measurement of serum uric acid, so we were not able to consider fluctuations in values. Although our research revealed a positive correlation between high serum uric acid levels and female infertility, the mechanism of action of uric acid has not been confirmed. More high-quality studies are needed in the future to confirm the findings of this study and to investigate whether controlling serum uric acid levels in women can improve female fertility.

## Conclusion

Our results indicate that there is a positive correlation between high serum uric acid levels and the risk of female infertility. Women with high uric acid levels are more likely to suffer from infertility. This study provides new ideas and references for improving female reproductive health and preventing infertility. However, more large-scale and high-quality research is needed in the future to confirm our conclusions and explore the physiological mechanisms involved.

## Supplementary Information

Supplementary Information.

## Data Availability

The datasets used for this study are publicly available from the National Center for Health Statistics website [https://www.cdc.gov/nchs/nhanes/index.htm].
