# Supplementary material for: Elevated serum uric acid is associated with infertility in women living in America
Source: Sci Rep. 2023 May 11;13:7687. doi: 10.1038/s41598-023-34702-x (PMC10173902; doi:10.1038/s41598-023-34702-x)
Supplement: Supplementary file 1 — Supplementary Information. [file 41598_2023_34702_MOESM1_ESM.pdf]

Elevated serum uric acid is associated with infertility in women living in America

Xiang Hong[#], Fanqi Zhao[#], Wei Wang, Jingying Wu, Xiaoqi Zhu, Bei Wang[*]

[1]Key Laboratory of Environmental Medicine and Engineering of Ministry of Education, Department of Epidemiology and Health Statistics, School of Public Health, Southeast University, Nanjing, Jiangsu, China

[#]These authors contributed equally to this work and should be considered co-first authors.

[*]**Corresponding author:** #87, Dingjiaqiao Road, Gulou District, Nanjing City, Jiangsu Province, China. 210009. Email: wangbeilxb@163.com

Supplementary material

Supplementary Table 1. The association between uric acid and infertility in the complete case

Supplementary Table 2. Stratified analysis of complete cases

Supplementary Table 1. The association between uric acid and infertility in the complete case

	Infertility	Control	Unadjusted OR (95%CI)	P-value	Adjusted OR (95%CI)[a]	P-value	Adjusted OR (95%CI)[b]	P-value
Uric acid (continuous variable)	295	1902	**1.24 (1.11, 1.37)**	**< 0.001**	**1.22 (1.1, 1.36)**	**< 0.001**	1.17 (0.97, 1.42)	0.102
Uric acid (categorical variable)								
2.4-5.7	249	1654	1.00 (1.00, 1.00)	1	1.00 (1.00, 1.00)	1	1.00 (1.00, 1.00)	1
<2.4	1	16	0.42 (0.05, 3.14)	0.395	0.38 (0.05, 2.9)	0.35	-	0.984
>5.7	45	232	1.29 (0.91, 1.82)	0.151	1.2 (0.84, 1.71)	0.319	0.82 (0.45, 1.5)	0.512
Uric acid (quartile)								
Q1 (1.6-3.7)	52	508	1.00 (1.00, 1.00)	1	1.00 (1.00, 1.00)	1	1.00 (1.00, 1.00)	1
Q2 (3.7-4.4)	67	503	1.3 (0.89, 1.91)	0.177	1.29 (0.88, 1.9)	0.198	1.18 (0.63, 2.2)	0.614
Q3 (4.4-5.1)	84	443	**1.85 (1.28, 2.68)**	**0.001**	**1.85 (1.27, 2.68)**	**0.001**	**2.1 (1.15, 3.84)**	**0.015**
Q4 (5.1-18.0)	92	448	**2.01 (1.4, 2.88)**	**< 0.001**	**1.98 (1.37, 2.88)**	**< 0.001**	**1.95 (1.02, 3.72)**	**0.045**

[a]Adjusted for age, race, marital status, smoking, alcohol, history of pregnancy, history of diabetes, history of hypertension.

[b]Adjusted for age, race, marital status, smoking, alcohol, history of pregnancy, history of diabetes, history of hypertension, fasting glucose, total cholesterol, creatinine-refrigerated serum, LDL-cholesterol, direct HDL-cholesterol, glycohemoglobin, BMI.

Supplementary Table 2. Stratified analysis of complete cases

Stratified factor	N	Q1	Q2<br>OR (95%CI)	P	Q3<br>OR (95%CI)	P	Q4<br>OR (95%CI)	P
Age (years)								
≤35	1135	ref	1.48 (0.52, 4.18)	0.463	**2.83 (1.02, 7.79)**	**0.045**	2.38 (0.83, 6.84)	0.108
>35	1062	ref	0.96 (0.42, 2.18)	0.921	1.62 (0.73, 3.61)	0.234	1.58 (0.65, 3.81)	0.313
Marital status								
Married/Living with Partner	1431	ref	1.09 (0.53, 2.28)	0.808	**2.32 (1.15, 4.65)**	**0.018**	1.73 (0.81, 3.7)	0.159
Widowed/Divorced/Separated	278	ref	2.02 (0.32, 12.6)	0.453	1.4 (0.22, 8.86)	0.721	1.69 (0.18, 16.1)	0.648
Never married	488	ref	2.14 (0.27, 16.74)	0.47	2.55 (0.3, 21.61)	0.39	**8.97 (1.01, 80.02)**	**0.049**
BMI								
<18.5	42	ref	-	-	-	-	-	-
18.5-24.9	614	ref	0.97 (0.31, 2.98)	0.951	**3.42 (1.15, 10.13)**	**0.026**	0.84 (0.14, 5.02)	0.846
25-29.9	548	ref	0.65 (0.12, 3.51)	0.619	2.55 (0.59, 11.06)	0.211	3.45 (0.76, 15.76)	0.11
≥30	979	ref	1.91 (0.63, 5.81)	0.255	2.15 (0.74, 6.23)	0.159	2.21 (0.77, 6.33)	0.139
Database year								
2013-2016	1266	ref	1.29 (0.52, 3.22)	0.584	1.89 (0.77, 4.62)	0.166	2 (0.77, 5.21)	0.154
2017-March 2020 pre-pandemic	931	ref	0.99 (0.39, 2.51)	0.987	2.27 (0.95, 5.44)	0.065	2.07 (0.79, 5.41)	0.139

BMI: body mass index.
